# Physiological impact of high-flow nasal cannula therapy on postextubation acute respiratory failure after pediatric cardiac surgery: a prospective observational study

**DOI:** 10.1186/s40560-017-0226-z

**Published:** 2017-06-06

**Authors:** Naohiro Shioji, Tatsuo Iwasaki, Tomoyuki Kanazawa, Kazuyoshi Shimizu, Tomohiko Suemori, Kentaro Sugimoto, Yasutoshi Kuroe, Hiroshi Morimatsu

**Affiliations:** Department of Anesthesiology and Resuscitology, Okayama University Hospital, 2-5-1 Shikatachou, Kitaku, Okayama, Okayama 700-0914 Japan

**Keywords:** Oxygen inhalation therapy, Respiratory insufficiency, Heart defects, Congenital

## Abstract

**Background:**

Reintubation after pediatric cardiac surgery is associated with a high rate of mortality. Therefore, adequate respiratory support for postextubation acute respiratory failure (ARF) is important. However, little is known about the physiological impact of high-flow nasal cannula (HFNC) therapy on ARF after pediatric cardiac surgery. Our working hypothesis was that HFNC therapy for postextubation ARF after pediatric cardiac surgery improves hemodynamic and respiratory parameters.

**Methods:**

This was a prospective observational study conducted at a single university hospital. Children less than 48 months of age who had postextubation ARF after cardiac surgery were included in this study. HFNC therapy was started immediately after diagnosis of postextubation ARF.

Data obtained just before starting HFNC therapy were used for pre-HFNC analysis, and data obtained 1 h after starting HFNC therapy were used for post-HFNC analysis. We compared hemodynamic and respiratory parameters between pre-HFNC and post-HFNC periods. The Wilcoxon signed-rank test was used to analyze these indices.

**Results:**

Twenty children were included in this study. The median age and body weight were 4.5 (2.3–14.0) months and 4.3 (3.1–7.1) kg, respectively. Respiratory rate (RR) significantly decreased from 43.5 (32.0–54.8) to 28.5 (21.0–40.5) breaths per minute (*p* = 0.0008) 1 h after the start of HFNC therapy. Systolic blood pressure also decreased from 87.5 (77.8–103.5) to 76.0 (70.3–85.0) mmHg (*p* = 0.003). Oxygen saturation, partial pressure of arterial carbon dioxide, heart rate, and lactate showed no remarkable changes. There was no adverse event caused by HFNC therapy.

**Conclusions:**

HFNC therapy improves the RR of patients who have postextubation ARF after pediatric cardiac surgery without any adverse events.

## Background

The rate of reintubation after pediatric cardiac surgery is about 6–9%, and it is associated with a high rate of mortality [1, 2]. Therefore, adequate respiratory support for postextubation acute respiratory failure (ARF) is important.

A high-flow nasal cannula (HFNC) is a respiratory support device that can deliver heated and humidified air and oxygen at a higher rate than the patient’s inspiratory flow rate [3]. It can provide precise fractional oxygen delivery and mild positive airway pressure, and it flushes the nasopharyngeal dead space, reducing airway resistance. Thus, an HFNC therapy reduces the patient’s work of breathing [4–7].

An HFNC is widely used for treatment of respiratory failure in children with bronchiolitis [8–10]. However, there has been only one study on the use of an HFNC after pediatric cardiac surgery [11]. There has been no report on the use of an HFNC for ARF after pediatric cardiac surgery. Hence, there is no information about the physiological effect of HFNC therapy on ARF after pediatric cardiac surgery. Our working hypothesis was that HFNC therapy for postextubation ARF after pediatric cardiac surgery improves hemodynamic and respiratory parameters.

## Methods

### Study design and patient population

This was a prospective observational study. The study period was 1 year (January 1, 2014, to December 31, 2014). The study was conducted in a tertiary teaching hospital that had 865 beds including 8 beds in the pediatric cardiac ICU. The pediatric cardiac ICU has approximately 400 admissions per year. Children less than 48 months of age who had postextubation ARF after pediatric cardiac surgery were included. There was no exclusion criterion. The definition of ARF is given in Table 1. We determined this definition by modifying the definition used in a previous report [12]. The use of accessory respiratory muscle was defined as intercostal retractions, sternal retractions, thoraco-abdominal dissociation, or nasal flaring.

**Table 1:** Definition of postextubation acute respiratory failure

Tachypnea	RR >50 breaths per minute (<1 year old) RR >40 breaths per minute (1–4 year old)
Hypoxemia	SaO_2_ <92% (total repair) SaO_2_ <75% (palliative operation)
Hypercapnia	PaCO_2_ >50 mmHg
Increased work of breathing	Using accessory respiratory muscle

Postextubation acute respiratory failure is defined by at least one of the criteria

*RR* respiratory rate, *PaCO*_*2*_ partial pressure of arterial carbon dioxide

In this study, we included patients with non-cyanotic heart disease and patients with cyanotic heart disease. We categorized the patients by the type of heart physiology (serial circulation vs. single ventricle) and did subgroup analysis.

### CPB management

We routinely do continuous ultrafiltration during cardiopulmonary bypass (CPB) and also do modified ultrafiltration just after the patient has been weaned from CPB. Continuous ultrafiltration and modified ultrafiltration were performed for all of the patients in this study.

### HFNC management

HFNC (Optiflow; Fisher and Paykel Healthcare Ltd., Auckland, New Zealand) therapy was started immediately after diagnosis of postextubation ARF. The flow was commenced at 2 L/kg/min. The inspiratory oxygen fraction (F_I_O_2_) was set to achieve target oxygen saturation (total repair, >92%; palliative operation, 75–85%). The temperature was set to 37° with a humidifier. The nasal cannula size was selected according to the weight and nasal size of the patient.

### Measurements

The outcome was physiological impact of HFNC therapy on postextubation ARF after pediatric cardiac surgery. Data for age, body weight, gender, risk adjustment for congenital heart surgery (RACHS-1) category [13], and diagnosis were collected prospectively. Bedside nurses collected data for respiratory rate (RR) and stored the data in an electronic healthcare record (EHR). Data for systolic blood pressure (SBP) and heart rate (HR) were stored in an EHR automatically by using an electrical system.

Oxygen saturation (SaO_2_), partial pressure of arterial carbon dioxide (PaCO_2_), and lactate were analyzed using a blood gas analysis apparatus (ABL 800, Radiometer Co., Copenhagen, Denmark), and these data were also stored in the EHR. Data collected just before starting HFNC therapy were used for pre-HFNC analysis, and data collected 1 h after starting HFNC therapy were used for post-HFNC analysis. We compared these hemodynamic and respiratory parameters in the pre-HFNC and post-HFNC periods.

### Statistical analysis

A previous study showed that the standard deviation of children’s RR was 15.4 [14]. We determined that a sample of 20 children was required to show power of 0.8 with an alpha error of 0.05 to detect 10 breaths per minute difference.

Continuous data were expressed as medians and their interquartile range (IQR) because of non-normal distribution. Categorical data were expressed as percentages. The Wilcoxon signed-rank test was used to compare physiological data. *P* values less than 0.05 were considered statistically significant. All statistical analyses were performed using statistical software (JMP® 11, SAS Institute Inc., Cary, NC, USA).

## Results

Baseline characteristics and cardiac diagnoses of the patients are shown in Table 2. Twenty children were included in the study. The median age and body weight were 4.5 (2.3–14.0) months and 4.3 (3.1–7.1) kg, respectively. Major cardiac diagnoses of the patients were hypoplastic left heart syndrome, atrioventricular defect, and ventricular septal defect. The reasons for performing HFNC therapy are shown in Table 3. The major reason for HFNC therapy was use of accessory respiratory muscle. The median settings of the HFNC were flow rate of 2.1 (1.6–3.3) L/kg/min and F_I_O_2_ of 0.55 (0.3-0.68). The median time to diagnosis of ARF after extubation was 4 (0.75–21) h.

**Table 2:** Patient baseline characteristics

Baseline characteristics	Median (IQR)
Age (month)	4.5 (2.8–10.0)
Body weight (kg)	4.0 (2.9–6.8)
Male gender (%)	80
RACHS-1	3 (2–3)
Palliative operation (%)	40
Mechanical ventilation (hours)	75 (9–288)
Cardiac diagnosis	*n* (%)
HLHS	5 (25)
CAVSD	4 (20)
VSD	3 (15)
TOF	2 (10)
Others (SA/SV, TGA, DORV, TAPVR, IAA, TA)	6 (30)
Cause of acute respiratory failure	*n* (%)
Atelectasis	5 (25)
Heart failure/fluid overload	4 (20)
Uncontrolled airway secretion	4 (20)
Upper airway obstruction	2 (10)
Airway bleeding	1 (5)
Hypoventilation (due to sedative drugs)	1 (5)
Weak cough	1 (5)
Unclassified	2 (10)
Outcomes	*n* (% or IQR)
HFNC failure, *n* (%)	1 (5)
24 h reintubation, *n* (%)	1 (5)
ICU length of stay (days)	11 (7.5–17)

*IQR* interquartile range, *RACHS-1* risk adjustment for congenital heart surgery, *HLHS* hypoplastic left heart syndrome, *CAVSD* complete atrioventricular septal defect, *VSD* ventricular septal defect, *TOF* tetralogy of fallot, *SA/SV* single atrium/single ventricle, *TGA* transposition of great arteries, *DORV* double outlet right ventricle, *TAPVR* total anomalous pulmonary venous return, *IAA* interruption of aortic arch, *TA* tricuspid atresia, *HFNC* high-flow nasal cannula

**Table 3:** Numbers of patients met criteria for acute respiratory failure

Reason	*n* (%)
Tachypnea	7 (35)
Hypoxemia	6 (30)
Hypercapnia	8 (40)
Using accessory respiratory muscle	20 (100)

Physiological outcome data are shown in Table 4. Among the hemodynamic variables, SBP significantly decreased from 87.5 (77.8–103.5) to 76.0 (70.3–85.0) mmHg (*p* = 0.003) after the start of HFNC therapy. There was no remarkable change in HR or lactate. Among the respiratory variables, RR significantly decreased from 43.5 (32.0–54.8) to 28.5 (21.0–40.5) breaths per minute (*p* = 0.0008) after the start of HFNC therapy. PaCO_2_ dropped, but the difference was not significant (46.4 vs. 46.0 mmHg, *p* = 0.05). SaO_2_ did not change after the start of HFNC therapy (92.9 vs. 95.1%, *p* = 0.15). The median duration of HFNC therapy was 44.0 (23.0–79.3) h. One (5%) of the patients was reintubated. Reintubation was performed for respiratory and cardiac reasons. There was no adverse event caused by HFNC therapy.

**Table 4:** Hemodynamic and respiratory parameters before and after high-flow nasal cannula therapy (*n* = 20)

Parameters	Pre-HFNC	Post-HFNC	*p* value
RR (breaths per minute)	43.5 (32.0–54.8)	28.5 (21.0–40.5)	0.0008
PaCO_2_ (mmHg)	46.4 (41.1–55.9)	46.0 (42.1–51.9)	0.05
SaO_2_ (%)	92.9 (77.5–97.1)	95.1 (3.0–98.9)	0.15
SBP (mmHg)	87.5 (77.8–103.5)	76.0 (70.3–85.0)	0.003
HR (beats per minute)	143.5 (111.3–155.8)	117.5 (109.5–143.0)	0.34
Lactate (mmol/l)	1.1 (0.5–1.7)	1.0 (0.8–1.5)	0.21

*HFNC* high-flow nasal cannula, *RR* respiratory rate, *PaCO*_*2*_ partial pressure of arterial carbon dioxide, *SaO*_*2*_ arterial oxygen saturation, *SBP* systolic blood pressure, *HR* heart rate

The results of subgroup analysis were shown in Tables 5 and 6. The single ventricle group included patients with hypoplastic left heart syndrome (*n* = 5), single atrium/single ventricle (*n* = 1), and tricuspid atresia (*n* = 1). In the single ventricle group, there were no significant differences in hemodynamics and respiratory parameters after the start of HFNC therapy. In the serial circulation group, RR and SBP dropped significantly after the start of HFNC therapy.

**Table 5:** Baseline characteristics of subgroup

Baseline characteristics	Serial circulation group (*n* = 13)	Single ventricle group (*n* = 7)
Age (month)	5 (1–7.5)	4 (3–25)
Body weight (kg)	4.1 (3.6–6.7)	5 (2.7–9.7)
Male gender (%)	10 (77)	4 (57)
RACHS-1	3 (2–3)	3 (3–6)
Mechanical ventilation (hours)	112 (22–268)	43 (16–119)

*RACHS-1* risk adjustment for congenital heart surgery

**Table 6:** Subgroup analysis: hemodynamic and respiratory parameters before and after high-flow nasal cannula therapy for serial circulation group (*n* = 13) and single ventricle group (*n* = 7)

	Serial circulation group (*n* = 13)			Single ventricle group (*n* = 7)		
Parameters	Pre-HFNC	Post-HFNC	*p* value	Pre-HFNC	Post-HFNC	*p* value
RR (breaths per minute)	43 (31.5–55)	25 (20.5–35.5)	0.003	44 (32–55)	34 (32–43)	0.2
PaCO_2_ (mmHg)	45.7 (41.1–53.2)	46.3 (42.7–48.2)	0.2	50.4 (38.1–61)	45.6 (38.6–57.7)	0.3
SaO_2_ (%)	95.4 (92.2–98.9)	98.7 (86.5–99.4)	0.5	69.3 (58.1–88.6)	80.6 (65.3–88.3)	0.2
SBP (mmHg)	95 (77–105)	76 (68.5–80)	0.007	85 (80–93)	80 (71–91)	0.3
HR (beats per minute)	150 (110–164)	115 (108–130)	0.08	138 (112–147)	143 (116–150)	0.4
Lactate (mmol/l)	1.1 (0.5–1.6)	0.9 (0.7–1.4)	0.4	1.1 (1.0–2.9)	1.1 (1.0–2.2)	0.5

*HFNC* high-flow nasal cannula, *RR* respiratory rate, *PaCO*_*2*_ partial pressure of arterial carbon dioxide, *SaO*_*2*_ arterial oxygen saturation, *SBP* systolic blood pressure, *HR* heart rate

## Discussion

The results of this study are valuable since we selected patients who had needed respiratory support because of ARF. There has been no other study about HFNC therapy for ARF after pediatric cardiac surgery. In addition, positive pressure induced by HFNC therapy may be deleterious in this specific patient cohort. Therefore, we selected these patients as a study target group to investigate the physiological impact of the use of an HFNC. Therefore, this is the first study that provides information about the physiological impact of the use of an HFNC for patients with ARF after pediatric surgery. Our results showed that HFNC therapy for postextubation ARF after pediatric cardiac surgery decreased SBP and RR in mixed serial circulation patients and single ventricle patients. This might be a beneficial effect of HFNC therapy for postextubation ARF after pediatric cardiac surgery. All of the patients in this study used accessory respiratory muscle. In this situation, reduced RR contributed to the reduction of work of breathing. A decrease in SBP might be caused by an improved respiratory state, which may reduce sympathetic nerve activity.

On the other hand, PaCO_2_, SaO_2_, Lactate, and HR did not change significantly. Increased end-expiratory lung volume caused by positive end expiratory pressure could decrease CO_2_ clearance [15]. However, the airway gas washout effect of HFNC therapy may improve PaCO_2_ [16, 17]. These opposite effects may be the reason for no significant difference in PaCO_2_. Subgroup analysis also showed that SaO_2_ was not improved in either the serial circulation group or single ventricle group. SaO_2_ could be influenced by gas exchange in the alveolus, pulmonary blood flow, and F_I_O_2_. Therefore, we could not provide a definitive conclusion about change of SaO_2_. Lactate represents adequate oxygen supply to peripheral tissues. Thus, lactate does not always have a direct relationship with respiratory state. That was the reason why there was no significant difference in lactate in either of the groups. In our study, HR showed a large variation. We guess this influenced our results of no significant difference in HR.

The subgroup analysis showed that the beneficial effect of HFNC therapy on RR was limited to serial circulation. However, these results of subgroup analysis were not powered for subgroup analysis and should be interpreted carefully. Thus, further study is needed to investigate the impact of HFNC therapy on single ventricle patients.

There has been one randomized controlled trial (RCT) using HFNC therapy after pediatric cardiac surgery. Testa et al. showed that the use of HFNC therapy improved partial pressure of arterial oxygen (PaO_2_) in children after cardiac surgery but that there was no impact on PaCO_2_ [11]. Our results are concordant with their results, showing that HFNC therapy has no impact on PaCO_2_. However, their study targeted children without ARF. In this situation, some children might not need respiratory support. The results must therefore be interpreted carefully.

In previous studies, rates of reintubation after pediatric cardiac surgery were 6–9% [1, 2]. In our study, only one (5%) of the 20 patients was reintubated because of failure of HFNC therapy. Our patients were selected patients at risk of reintubation. Despite the selection of such patients, the reintubation rate was lower than that in past studies. Schibler et al. showed in their retrospective study that HFNC therapy might reduce the need for intubation in infants with bronchiolitis [18]. Further study is needed to confirm the effectiveness of HFNC therapy for preventing reintubation after pediatric cardiac surgery.

### Limitations

This study has some limitations. Firstly, we compared hemodynamic and respiratory parameters before and after HFNC therapy in the same patients. There was no control group in this study. We cannot rule out the possibility of improvement in RR without HFNC therapy. ARF is a critical event for patients after pediatric cardiac surgery. HFNC therapy for ARF has been used in our daily practice. Therefore, it was not clinically practical and ethically permissible to set a control group. Thus, we carried out observational study instead of an RCT or crossover study. There have been no available data about the use of an HFNC therapy for ARF after pediatric surgery. Our results for selected patients may be valuable for a future RCT.

Secondly, we included patients with various conditions (age, cyanosis, type of operation), and our definition of ARF is specific in our institute. It is therefore difficult to generalize that HFNC therapy is beneficial for ARF after pediatric cardiac surgery.

## Conclusions

In conclusion, HFNC therapy improves the respiratory state of patients with postextubation ARF after pediatric cardiac surgery by reducing RR. The HFNC may be a useful device in postextubation ARF after pediatric cardiac surgery.

## References

[1] Gupta P, Rettiganti M, Gossett JM, Yeh JC, Jeffries HE, Rice TB, Wetzel RCJ (2016). Risk factors for mechanical ventilation and reintubation after pediatric heart surgery. Thorac Cardiovasc Surg.

[2] Gaies M, Tabbutt S, Schwartz SM, Bird GL, Alten JA, Shekerdemian LS, Klugman D, Thiagarajan RR, Gaynor JW, Jacobs JP, Nicolson SC, Donohue JE, Yu S, Pasquali SK, Cooper DS (2015). Clinical epidemiology of extubation failure in the pediatric cardiac ICU: a report from the Pediatric Cardiac Critical are Consortium. Pediatr Crit Care Med.

[3] Lee JH, Rehder KJ, Williford L, Cheifetz IM, Turner DA (2013). Use of high flow nasal cannula in critically ill infants, children, and adults: a critical review of the literature. Intensive Care Med.

[4] Dewan NA, Bell CW (1994). Effect of low flow and high flow oxygen delivery on exercise tolerance and sensation of dyspnea. A study comparing the transtracheal catheter and nasal prongs. Chest.

[5] Shepard JW, Burger CD (1990). Nasal and oral flow-volume loops in normal subjects and patients with obstructive sleep apnea. Am Rev Respir Dis.

[6] Rubin S, Ghuman A, Deakers T, Khemani R, Ross P, Newth CJ (2014). Effort of breathing in children receiving high-flow nasal cannula. Pediatr Crit Care Med.

[7] Nishimura M (2015). High-flow nasal cannula oxygen therapy in adults. J Intensive Care.

[8] Milési C, Boubal M, Jacquot A, Baleine J, Durand S, Odena MP, Cambonie G (2014). High-flow nasal cannula: recommendations for daily practice in pediatrics. Ann Intensive Care.

[9] Hough JL, Pham TM, Schibler A (2014). Physiologic effect of high-flow nasal cannula in infants with bronchiolitis. Pediatr Crit Care Med.

[10] Beggs S, Wong ZH, Kaul S, Ogden KJ, Walters JA (2014). High-flow nasal cannula therapy for infants with bronchiolitis. Cochrane Database Syst Rev.

[11] Testa G, Iodice F, Ricci Z, Vitale V, De Razza F, Haiberger R, Iacoella C, Conti G, Cogo P (2014). Comparative evaluation of high-flow nasal cannula and conventional oxygen therapy in paediatric cardiac surgical patients: a randomized controlled trial. Interact Cardiovasc Thorac Surg.

[12] Muñoz-Bonet JI, Flor-Macián EM, Brines J, Roselló-Millet PM, Cruz Llopis M, López-Prats JL, Castillo S (2010). Predictive factors for the outcome of noninvasive ventilation in pediatric acute respiratory failure. Pediatr Crit Care Med.

[13] Jenkins KJ, Gauvreau K, Newburger JW, Spray TL, Moller JH, Iezzoni LI (2002). Consensus-based method for risk adjustment for surgery for congenital heart disease. J Thorac Cardiovasc Surg.

[14] Abadesso C, Nunes P, Silvestre C, Matias E, Loureiro H, Almeida H (2012). Non-invasive ventilation in acute respiratory failure in children. Pediatr Rep.

[15] Ten Brink F, Duke T, Evans J (2013). High-flow nasal prong oxygen therapy or nasopharyngeal continuous positive airway pressure for children with moderate-to-severe respiratory distress?. Pediatr Crit Care Med.

[16] Levy SD, Alladina JW, Hibbert KA, Harris RS, Bajwa EK, Hess DR (2016). High-flow oxygen therapy and other inhaled therapies in intensive care units. Lancet.

[17] Corley A, Caruana LR, Barnett AG, Tronstad O, Fraser JF (2011). Oxygen delivery through high-flow nasal cannulae increase end-expiratory lung volume and reduce respiratory rate in post-cardiac surgical patients. Br J Anaesth.

[18] Schibler A, Pham TM, Dunster KR, Foster K, Barlow A, Gibbons K, Hough JL (2011). Reduced intubation rates for infants after introduction of high-flow nasal prong oxygen delivery. Intensive Care Med.

